# Use of “Hidden in Plain Sight” de-identification methodology in electronic healthcare data provides minimal risk of misidentification: Results from the iCAIRD Safe Haven Artificial Intelligence Platform.

**DOI:** 10.23889/ijpds.v7i3.2023

**Published:** 2022-08-25

**Authors:** Luke Farrow, Katie Wilde, Jaroslaw Dymiter, Moragh Boyle, Alexander Weir, James Blackwood, Charlie Mayor, David Harrison, Lesley Anderson

**Affiliations:** 1 Aberdeen Centre for Health Data Science, University of Aberdeen; 2 Grampian Data Safe Haven, University of Aberdeen; 3 Canon Medical Research Europe; 4 NHS Greater Glasgow and Clyde; 5 School of Medicine, University of St Andrews

## Objectives

To determine the risk of misidentification when using a “Hidden In Plain Sight (HIPS)” Named Entity Recognition (NER) de-identification methodology applied to Scottish healthcare data within The Industrial Centre for Artificial Intelligence Research in Digital Diagnostics (iCAIRD) Safe Haven Artificial Intelligence Platform (SHAIP).

## Approach

Rather than the traditional redaction of potential identifiable information in routinely collected healthcare data, our HIPS methodology utilises an NER “find and replace” approach to de-identification that keeps the structure of text intact. This ensures that context is maintained, key to the interpretation of free text information and potential Artificial Intelligence applications.

To our knowledge these methods have been previously untested on Scottish healthcare data. We therefore performed assessment of this approach in terms of potential risk of misidentification using HIPS on structured Scottish data deployed in SHAIP as part of the iCAIRD programme.

## Results

Five individual cohorts, with a total of 169,964 patients were included. For each cohort the HIPS approach was applied, and then compared to actual patient information from within the same region, in order to determine the risk of misidentification. The following fields were included: Forename, Surname, Previous Name, Gender, Date of Birth (DOB), and Postcode.

Across the five cohorts and varying combinations of identifiable data fields there were a total of 94 instances of potential misidentification (0.06%). 85/94 (90.4%) of these were for the combination of Gender, Date of Birth and Postcode. Across the five cohorts there were only 3 instances (0.002%) of Forename/Surname/DOB, and 5 instances (0.003%) of Forename/Surname/Postcode potential misidentification amongst the 169,964 patients.

## Conclusion

The iCAIRD NER HIPS Methodology provides an acceptably low misidentification rate. Further work is now required to determine the recall and precision rates. Benefits of this approach include retaining the structure of free text, as well as reducing the ability to detect any potential leaked identifiable data.

